# Star fruit nephrotoxicity: a case series and literature review

**DOI:** 10.1186/s12882-018-1084-1

**Published:** 2018-10-22

**Authors:** Dilushi Rowena Wijayaratne, V. Bavanthan, M. V. C. de Silva, A. L. M. Nazar, Eranga S. Wijewickrama

**Affiliations:** 1 Univeristy Medical Unit, National Hospital of Sri Lanka, Colombo, Sri Lanka; 2 Nephrology, Dialysis and Transplant Unit, National Hospital of Sri Lanka, Colombo, Sri Lanka; 3 Department of Pathology, Faculty of Medicine, University of Colombo, Colombo, Sri Lanka; 4 Department of Clinical Medicine, Faculty of Medicine, University of Colombo, Colombo, Sri Lanka

**Keywords:** Star fruit, Oxalate, Acute kidney injury

## Abstract

**Background:**

Star fruit is a popular medicinal fruit in the tropics. Its hypoglycaemic properties are considered useful in achieving glycaemic control in diabetes. Star fruit induced nephrotoxicity is a rare cause of acute kidney injury in individuals with both normal and reduced baseline renal function. We present three cases of acute kidney injury due to star fruit nephrotoxicity from Sri Lanka, and discuss the published literature on this topic.

**Case presentation:**

Three Sri Lankan patients, all with a background of diabetes, presented to us with acute nausea and anorexia following recent consumption of star fruit. Two patients complained of diarrhoea and one patient complained of intractable hiccoughs. They all had elevated serum creatinine on admission. Two were known to have normal baseline serum creatinine levels. On renal biopsy two had evidence of oxalate crystal deposition. One did not show crystal deposition but had acute interstitial nephritis for which no alternate cause could be identified. Two were treated with short courses of prednisolone and two required acute haemodialysis. All recovered renal function, with both patients with known baselines approaching their premorbid serum creatinine levels.

**Conclusion:**

Consumption of star fruit, especially on an empty stomach or in a state of dehydration may precipitate acute kidney injury. A history of star fruit ingestion must be actively looked for in patients presenting with unexplained acute kidney injury. The use of star fruit as a therapy for diabetes should be discouraged.

## Background

Star fruit (*Averrhoa carambola*) is a popular fruit in Asian tropics. It is valued for its medicinal and nutritional properties [1]. Its hypoglycaemic effects are considered to be particularly beneficial in patients with diabetes mellitus, and it has been promoted as a traditional remedy for diabetes [2]. However, excessive consumption of star fruit has been associated with the development of oxalate nephropathy in patients with both normal and abnormal baseline renal function [3, 4]. Star fruit induced oxalate nephropathy remains an under-recognized cause for both acute and chronic kidney disease. We report three cases of acute kidney injury following ingestion of star fruit and discuss the literature related to star fruit toxicity and renal disease.

## Case presentation

### Case 1

A 52-year-old male with a five-year history of type 2 diabetes mellitus presented with loose stools, abdominal pain and reduced urine output for two days. He had ingested of 200 ml of homemade star fruit juice made from four whole star fruits a few hours prior to the onset of symptoms. Notably he complained of intractable hiccoughs. His serum creatinine three months prior to the presentation had been 0.7 mg/dl. On admission he was mildly dehydrated and had a blood pressure of 140/90 mmHg. There was no evidence of diabetic retinopathy. Investigations revealed the following: haemoglobin- 13.5 g/dl, white cell count – 17, 840/ cumm (Neutrophils 79%, Lymphocytes- 10%, Eosinophils-0%), platelets 345,000/ cumm, serum creatinine 4.5 mg/dl, serum potassium 5.3 mmol/l, serum sodium 138 mmol/l, C – reactive protein- 164 mg/l. The urine sediment was bland with no proteinuria. His Anti-nuclear antibody (ANA) titre, Anti-streptolysin O titre (ASOT), Hepatitis B, C serology, Antineutrophil cytoplasm antibody (ANCA) titre, and Complement 3 (C3) and Complement 4 (C4) levels were normal. Renal ultrasound showed normal kidneys with preserved cortico-medullary demarcation. Urine and blood cultures were sterile. He was commenced on intravenous cefotaxime for suspected sepsis. By day 5 of illness serum creatinine rose to 9 mg/dl leading to the initiation of haemodialysis.

Renal biopsy was done on the sixth day. This showed ten glomeruli, one of which was sclerosed, the others being normal. Some of the tubules showed oxalate crystals associated with acute tubular epithelial injury and evidence of regeneration. Patchy tubular atrophy was seen. The interstitium was oedematous and infiltrated by a moderate inflammatory infiltrate comprising lymphocytes, plasma cells, eosinophils and neutrophils. Mild interstitial fibrosis was seen. A diagnosis of star fruit induced oxalate nephropathy was made.

He required haemodialysis only once and was discharged on the seventh day with a falling serum creatinine. Serum creatinine three months later had stabilized at baseline levels.

### Case 2

A 65-year-old male with a history of type 2 diabetes mellitus and hypertension presented with poor appetite, poor sleep, nausea and dyspeptic symptoms for five days. His urine output had been normal. On examination he was afebrile. His blood pressure was 120/70 mmHg. His laboratory investigations on admission revealed a serum creatinine of 7.3 mg/dl and serum K of 5.9 mmol/l. His serum creatinine done seven months before had been 1.2 mg/dl. Urinalysis was bland with no proteinuria. Serum ANA, ANCA, C3/C4, and Hepatitis B and C serology were normal.

He had not taken any prescription or over the counter medications in the recent past except for his usual anti diabetic medications. On direct questioning he admitted eating three fruits of star fruit immediately prior to the onset of symptoms. A clinical diagnosis of acute star fruit nephrotoxicity was made.

His serum creatinine remained static despite good urine output. A renal biopsy was performed due to the delay in recovery. It revealed acute tubulo-interstitial nephritis without oxalate deposition. Prednisolone was started at 30 mg daily. His serum creatinine subsequently improved and 10 months later had reduced to 1.4 mg/dl.

### Case 3

A 57-year-old male with a history of hypertension and type 2 diabetes mellitus was admitted with loss of appetite, nausea and diarrhoea. On examination he was afebrile with a blood pressure of 140/90 mmHg and background diabetic retinopathy. His serum creatinine was 13.16 mg/dl on admission with serum potassium of 4. 8 mmol/l. His urinalysis revealed 8–10 pus cells and 35–40 red cells per high power field without any casts or proteinuria. His ANA, ASOT, Hepatitis B, C serology, ANCA and C3/C4 were normal. His renal ultrasonography showed normal sized kidneys with multiple calculi in the upper and middle calyces of the right kidney with cortical thinning and slightly increased cortical echogenicity of the left kidney.

On direct questioning he revealed having consumed on average one star fruit daily over the preceding one year with increased consumption to three fruits per day over the preceding one month.

**Fig. 1:**
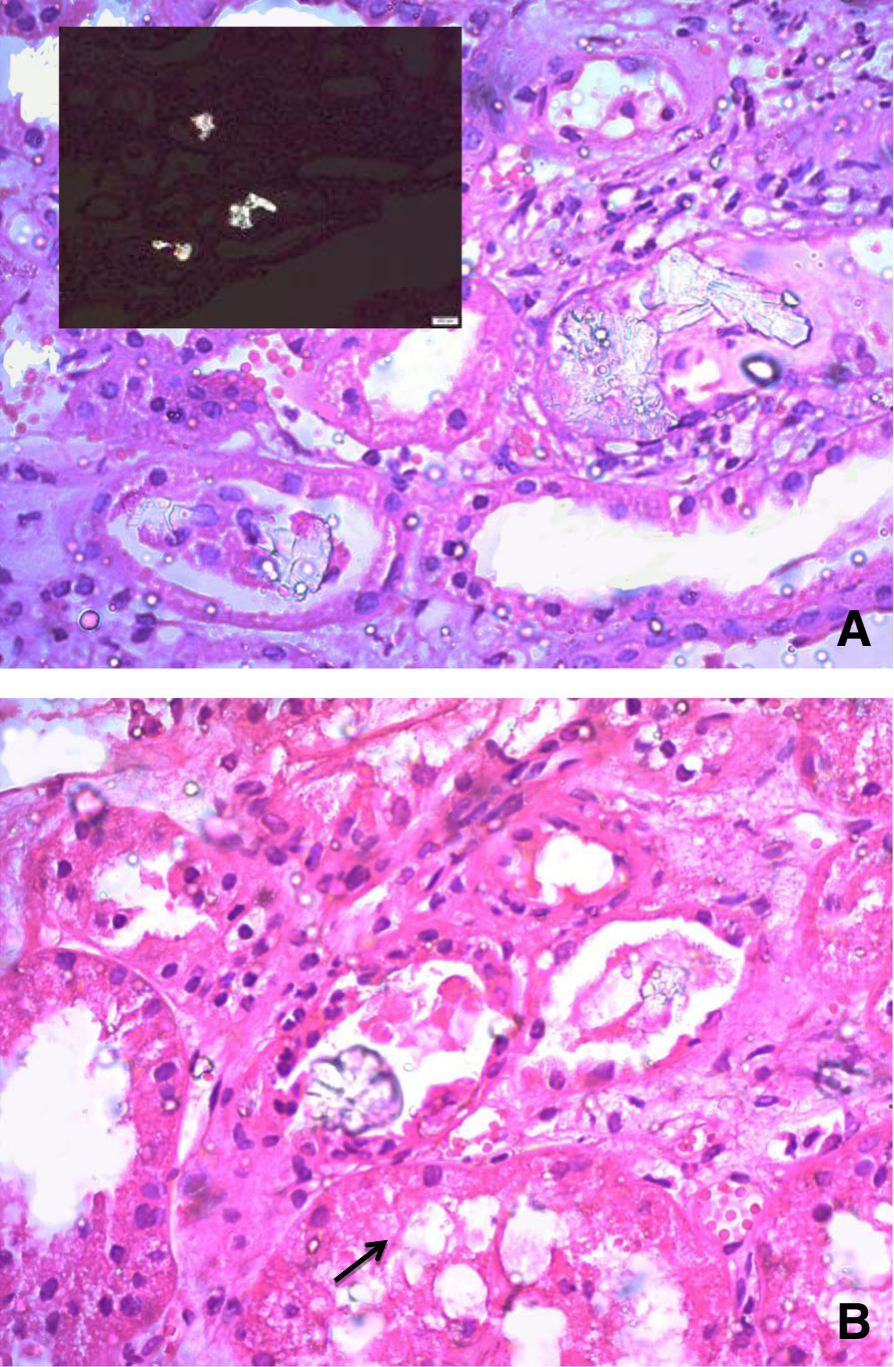
**a** Renal biopsy specimen of Case 3 showing intratubular oxalate crystals. Insert shows oxalate crystals under polarized light. (Haematoxylin & Eosin stain, X400). **b** Renal biopsy specimen of same patient showing focal attenuation of tubular epithelial cells with desquamation of cells. The tubule in the lower center shows cytoplasmic swelling and vacuolation, and intraluminal oxalate crystals (black arrow)

He underwent several sessions of haemodialysis. A renal biopsy was performed which revealed tubular injury in the presence of oxalate crystals within the tubular lumina (Fig. 1). There was mild tubular atrophy and mild focal interstitial fibrosis. Some glomeruli were enlarged. Three glomeruli showed increase in mesangial matrix. Few glomerular capillaries showed thickened basement membranes Appearances were compatible with oxalate nephropathy occurring in the background of early diabetic nephropathy.

He was started on prednisolone 30 mg daily. His serum creatinine subsequently improved and had reduced to 2.98 mg/dl two months after the initial admission.

## Discussion

*Averrhoa carambola* (star fruit), a member of the *Oxalidaceae* family, is rich in oxalic acid. The content of oxalate varies according to the type of star fruit, being higher in the sour type than in the sweet [5]. Total consumption is often higher when consumed as a juice than as a single whole fruit. The oxalic acid content of fresh juice was measured by Chen et al. as 202 mg/dl in fresh sweet juice and 829 mg/dl in fresh sour juice [6].Commercial preparations of star fruit juice are processed by pickling and diluting procedures which lower oxalate concentrations. In comparison homemade and medicinal preparations of pure sour star fruit juice often have high concentrations of oxalate [6].

In the gastro intestinal tract ingested oxalates complex with intraluminal calcium and magnesium to form insoluble complexes, which prevent them from getting absorbed [6]. When consumed in large amounts, especially in the fasting state, more free oxalates remain available for absorption into the circulation. Circulating free oxalates are filtered through the kidneys where they precipitate into calcium oxalate crystals and mediate renal damage by means of tubular obstruction. The formation of highly concentrated urine in the dehydrated state predisposes to crystal deposition. Typical histological findings include intraluminal and intraepithelial deposition of colourless oxalate crystals. When examined under polarized light these crystals exhibit a pattern of birefringence including all colours of the rainbow. Crystals appear blue in haematoxylin-eosin stain and black in von Kossa’s stain. With transmission electron microscopy, oxalate crystals appear mainly as long needles with blunt edges [7]. Some studies have shown a high serum oxalate level to be associated with increased apoptosis of renal epithelial cells which contributes to nephrotoxicity [8].

As yet there is no described toxic dose of star fruit juice. Toxicity is affected by the type of star fruit preparation as well as patient hydration and the fasting/ feeding state. Many patients with star fruit toxicity present within hours of ingestion with gastrointestinal symptoms such as vomiting and abdominal pain. These are suspected to be due to direct corrosive effects of dietary oxalates rather than systemic effects [9]. This may be followed by a reduction in urine output in patients who develop acute kidney injury. It has been suggested that the accumulation of the toxin caramboxin in patients with renal impairment results in the neuropsychiatric manifestations of star fruit toxicity. These include hiccoughs, confusion, insomnia and seizures [10]. One of our patients had intractable hiccoughs on presentation, a possible clue to the underlying diagnosis.

There are many cases of star fruit toxicity reported in the literature (Table 1) [3, 4, 6, 8, 10–35]. The toxic effects of star fruit were first described among patients with preceding renal impairment and were characterized by prominent neurotoxic features [4, 10, 11]. More recently, a total of 27 cases of acute kidney injury in patients with normal renal functions have been reported [6, 8, 29, 31–34]. The apparent increase in the reports of acute nephrotoxicity may reflect increasing awareness of the condition. Specific details about the clinical presentation and management of those with normal baseline creatinine were available for twelve of these cases. Neurotoxic features were seen in six out of twelve of these patients. While patients with underlying chronic kidney disease presented with toxic features after consumption of as little as half a star fruit, in those with normal renal function the minimal reported toxic doses were 4–6 star fruits or 300 ml of star fruit juice. All nine renal biopsies which had been done in previous studies, (two with background chronic kidney disease) showed evidence of oxalate deposition in renal tissue. Out of those twelve patients with normal baseline creatinine three required renal replacement therapy for treatment of acute kidney injury. Recovery was the norm. Only three of the 27 cases with normal renal function progressed to chronic kidney disease, with one becoming dialysis dependent.

**Table 1:** Summary of published case reports/ case series of star fruit neurotoxicity and nephrotoxicity

Year	Author	Country	Number of patients	Baseline renal function	Neurotoxicity	Renal biopsy	Treatment and outcome	Presence of diabetes	Type and dose of star fruit
1998	Neto et al. [10]	Brazil	6	On HD	+	N/A	HD One death	N/A	Ingestion of 2–3 fruits or 150–200 ml of the fruit juice
2000	Chang et al. [11]	Taiwan	20	15 on HD 4 on CAPD 1 SCr 6.4 mg/dl	+	N/A	Routine RRT +/− emergency HD 8 deaths including one patient who was pre-dialytic (out of 10 with impaired consciousness)	7/20	All patients who died had eaten one to two fresh fruits.
**2001**	**Chen et al** [6]	**Taiwan**	**2**	**Normal**	**–**	**Acute Oxalate Nephropathy (2/2)**	**HD. Recovered renal function**	**0/2**	**1.6 L and 3 L sour carombola juice**
2002	Chang et al. [12]	Taiwan	1	CKD (Scr 4.3 mg/dl)	+	N/A	HD. Recovered	1/1	2 star fruits
2002	Yap et al. [13] (from review)	Taiwan	3	CKD	+	N/A	HD 2 recovered. 1 died	N/A	N/A
2003	Neto et al.. [4]	Brazil	32	20 on HD 8 on PD 4 CKD on supportive care.	+	N/A	HD (conventional, daily), CRRT, IPD, symptomatic. 7 deaths	8/32	Half a fruit to 10 fruits
2003	Tse et al. [14]	Hong Kong	7	4 on CAPD 1 on HD 2 CKD- Predialyitic	+	N/A	3 intensive dialysis (PD/HD) PD and HD initiated in pre-dialytic All recovered	3/7	N/A
2004	Chang and Yeh [15]	Taiwan	1	HD	+	N/A	HD, phenytoin	1/1	Recovery
2005	Chen et al. [16]	Taiwan	1	CKD- predialytic	+	N/A	Two HD, followed by HP- recovered	1/1	3 star fruits
2005	Tsai et al... [17]	Taiwan	2	CKD- predialytic	+	N/A	HD, recovered	N/A	2–3 fresh fruits
2006	Niticharoenpong K et al..... [18]	Thailand	1	CKD-predialytic	+	Oxalate nephropathy	HD, partial renal recovery	N/A	N/A
2006	Wang et al. [19]	Taiwan	1	CKD- predialytic	+	N/A	HD, death	0/1	3 star fruits
2007	Wu et al. [20]	Taiwan	2	1 on HD 1 CKD V (predialytic)	+	N/A	HD followed by HP, both survived on chronic HD	1/2	1–2 star fruits
2008	Marín-Restrepo et al. [21]	Colombia	1	On HD	+	N/A	HD, recovered	N/A	N/A
2009	Chan et al. [22]	Hong Kong	1	CKD (eGFR 15 ml/min-MDRD)	+	N/A	Charcoal haemoperfusion, continuous haemofiltration, recovered eGFR 11 ml/min- MDRD)	1	2 star fruits
2009	Herbland et al. [23]	France	6	CKD	+	N/A	Two patients died Four recovered after standard dialysis, hemofiltration, and diafiltration	N/A	N/A
**2009**	**Neto et al..** [3]	**Brazil**	**5**	**Normal (S. Cr 79.5–106.2 micromol/l)**	**+**	**Acute oxalate nephropathy (2/2)**	**Full recovery with symptomatic care**	**N/A**	**12–15 fruits, 0.3- 1 l pure juice**
2009	Signaté A et al. [24]	France	2	CKD-predialytic	+	N/A	Recovered with haemofiltration	N/A	N/A
2010	Auxiliadora- Martins et al..... [25]	Brazil	1	CKD –pre-dialytic	+	N/A	HD. Died	0/1	Star fruit juice
2010	Chen et al. [26]	Taiwan	1	CKD (Serum creatinine 1.45 mg/dl)	+	N/A	HD for neurotoxicity, (Serum creatinine 1.8 mg/dl) Recovered	1/1	1 fresh fruit
2010	Moreira et al. [27]	Brazil	1	CKD	+	N/A	HD. Died	N/A	N/A
2011	Wu et al. [28]	Taiwan	2	1 on HD 1 CKD V – predialytic	+	N/A	SLEDD-f + charcoal HP. Both remained comatose. One died	½	1–2 star fruits
**2011**	**Su YJ** [29]	**Taiwan**	**1**	**Normal**	**–**	**Acute oxalate nephropathy**	**Supportive care. Recovered to serum creatinine 0.92 mg/dl**	**0/1**	**1 l pure juice**
2012	Alessio-Alves et Al [30]	Brazil	1	On HD	+	N/A	HD. Recovered	0/1	Star fruit
**2014**	**Scaranello et al **[8]	**Brazil**	**1**	**Normal**	**–**	**N/A**	**HD. Recovered renal function**	**N/A**	**N/A**
**2014**	**Ananna et al** [31]	**Banglad-esh**	**1**	**Normal**	**+**	**Acute tubular necrosis. Crystalline deposits**	**Supportive. Full recovery**	**1/1**	**Half a kilogram of starfruit**
**2015**	**Abeysekera et al** [32]	**Sri Lanka**	**2**	**Normal (1), CKD- serum creatinine 133 micromol/l (eGFR 50.6 ml/min) (1)**	**–**	**Oxalate nephropathy seen in both. Acute interstitial nephritis (1) Acute on chronic interstitial nephritis (1)**	**Prednisolone in patient with acute interstitial nephritis. Renal function recovered to baseline in both**	**2/2**	**4–6 star fruits (Patient CKD – on a background of regular consumption)**
**2015**	**Ananna et al** [33]	**Banglad-esh**	**20**	**Normal (15),** **CKD (5)**	**+**	**N/A**	**HD (5). Of 3 with normal baseline- 3 developed CKD, 1 HD dependent.** **Of baseline CKD- 1 became HD dependent**	**11/20**	**1–6 star fruits, 150 ml/500 ml juice**
**2017**	**Molina et al** [34]	**Peru**	**1**	**?normal**	**+**	**N/A**	**Urinary alkalization with potassium citrate. SCr recovered to 1.4 mg/dl**	**1/1**	**2–3 glasses of juice**
***2017***	***Wijayaratne*** **et al**	***Sri Lanka***	***3***	***Normal (2), unkown(1)***	***+***	***Oxalate nephropathy (2) Acute interstitial nephritis (1)***	***HD (2). Prednisolone given to two patients. Both with normal baseline creatinine recovered to baseline.***	***3/3***	***3–4 star fruits. In one case recent increase in consumption to 3/day on a background of 1 daily***

Bold- Background normal renal function

*CKD* chronic kidney disease, *HD* haemodialysis, *HP* Haemoperfusion, *CRRT* continuous renal replacement therapy, *IPD* intermittent peritoneal dialysis, *CAPD* continuous ambulatory peritoneal dialysis, SLEDD-f slow low efficiency daily diafiltration, *N/A* not available, + − present, −-absent

A case of chronic kidney disease attributable to frequent ingestion of star fruit was recently reported from Sri Lanka [32]. It has been suggested that chronic kidney disease may result from calcium oxalate induced interstitial inflammation, fibrosis and nephron loss. Two of our patients had normal premorbid serum creatinine which returned to baseline with time, indicative of pure acute kidney injury. The third had evidence of concomitant diabetic nephropathy on renal biopsy. In our series two patients had oxalate crystals while one had only features of acute interstitial nephritis. This highlights the need for obtaining a history of ingestion of potential nephrotoxins in a patient with unexplained acute kidney injury. It is interesting to note that both previous cases reported from Sri Lanka were also diabetic patients [32]. This may reflect the common use of star fruit among diabetics in Sri Lanka as a remedy to reduce the blood glucose levels or alternatively suggest an increased susceptibility in this group. The prevalence of diabetes as a comorbidity is 42.1% among reported cases in the literature. However, data is inadequate to comment on the prevalence of diabetes in those with normal baseline renal function. The fat malabsorption seen in chronic pancreatitis (a cause of diabetes) and diabetic gastroentoropathy (a complication of diabetes) may be associated with acute oxalate nephropathy [36, 37]. The free fatty acids that remain unabsorbed in the colonic lumen bind to calcium ions and prevent the precipitation of oxalate. Free oxalate can be absorbed in to the bloodstream and filtered in the kidney, as described above. The three patients in our series did not have clinical features of malabsorption. However, it would be interesting to study if subclinical degrees of malabsorption in diabetic patients predispose to oxalate nephropathy in the presence of an acute oxalate load.

There is no specific treatment for star fruit induced acute kidney injury. In those who require renal replacement therapy haemodialysis and haemoperfusion are preferred. Based on experience in the use of haemodialysis in the setting of primary hyperoxaluria, these methods may benefit patients by increasing the clearance of oxalate, apart from the removal of uremic toxins [38]. None of the studies described the use of haemodialysis for the sake of removal of oxalate per se without any other nephrological indications. However, it has been suggested that haemodialysis should be performed early if the disturbance in the conscious level of the patient is suspected to be directly related to star fruit intoxication rather than due to the resultant renal impairment [12].Peritoneal dialysis has been shown to be of no benefit, especially in patients with neurological features [4]. Though many nephrologists use steroids for the management of acute oxalate nephropathy, there are no studies to support this, and spontaneous recovery is the rule. Recently the effect of N- acetyl cysteine (NAC) on star fruit induced acute kidney injury was studied in animal models. The results suggest that NAC may attenuate renal dysfunction by means of reducing oxidative stress, oxaluria and inflammation [39].

## Conclusion

Star fruit nephrotoxicity must be considered in any individual developing unexplained acute kidney injury. The history is the key to reach a diagnosis early. As yet, there are no proven specific therapies and management is supportive. It is essential to prevent star fruit nephrotoxicity by educating the public and especially diabetics to avoid consuming star fruit, especially on an empty stomach or in a dehydrated state. Further studies need to be done to identify the dose and type of star fruit, which could lead to nephrotoxicity. In the interim the use of star fruit, specifically as a therapy to achieve better glycaemic control in diabetes, should be discouraged.

## Abbreviations

ANA: Antinuclear antibody
ANCA: Anti-neutrophil cytoplasm antibody
ASOT: Anti-streptolysin O titre
C3: Complement 3
C4: Complement 4
cumm: Cubic millimitre
dl: Decilitre
g: Gram
l: Litre
mg: Milligram
ml: Millilitre
mmHg: Millimetres of mercury
mmol: Millimole
NAC: N- acetylcysteine
